# Can discourse processing performance serve as an early marker of Alzheimer’s disease and mild cognitive impairment? A systematic review of text comprehension

**DOI:** 10.1007/s10433-021-00619-5

**Published:** 2021-04-20

**Authors:** Eesha Kokje, Simge Celik, Hans-Werner Wahl, Christiane von Stutterheim

**Affiliations:** 1 Network Aging Research, Heidelberg University, Bergheimer Strasse 20, 69115 Heidelberg, Germany; 2 Institut für Deutsch als Fremdsprachenphilologie, Heidelberg University, Heidelberg, Germany

**Keywords:** Discourse, Alzheimer’s disease, Mild cognitive impairment, Language, Comprehension

## Abstract

A number of linguistic and cognitive deficits have been reported during the course of Alzheimer’s disease (AD) and its preceding stage of mild cognitive impairment (MCI), with some deficits appearing years before onset of clinical symptoms. It continues to be a critical task to identify tools that may serve as an early marker of pathology that are also reliably able to distinguish AD from normal ageing. Given the limited success of classic psychometric cognitive testing, a novel approach in assessment is warranted. A potentially sensitive assessment paradigm is discourse processing. The aim of this review was to synthesize original research studies investigating comprehension of discourse in AD and MCI, and to evaluate the potential of this paradigm as a promising avenue for further research. A literature search targeting studies with AD or MCI groups over 60 years of age was conducted in PubMed, Web of Science, and PsycINFO databases. Eight articles with good quality were included in the review. Six measures of discourse comprehension—naming latency, summary, lesson, main idea, proportion of inferential clauses, true/false questions—were identified. All eight studies reported significant deficits in discourse comprehension in AD and MCI groups on five of the six measures, when compared to cognitively healthy older adults. Mixed results were observed for associations with commonly used cognitive measures. Given the consistent findings for discourse comprehension measures across all studies, we strongly recommend further research on its early predictive potential, and discuss different avenues for research.

**Supplementary Information:**

The online version contains supplementary material available at 10.1007/s10433-021-00619-5.

## Introduction

As life expectancy continues to increase, the ageing population continues to grow in number, and so does the prevalence and incidence of age-related disorders. Dementia is one of the most common age-related disorders, and is a major cause of concern worldwide due to its untreatable nature. As of 2018, an estimated 50 million people worldwide live with dementia, with the number expected to be over 152 million by the year 2050 (Patterson 2018). Alzheimer’s disease (AD) is the most common type of dementia, accounting for an estimated 60% to 80% of the cases. It results in progressive cognitive and functional decline, which is irreversible, and begins before clinical onset of AD. The clinical manifestation of AD is preceded by a transitional stage of mild cognitive impairment (MCI), which has received considerable attention as a target stage for early detection and interventions.

The long preclinical stage of AD is marked by irreversible neuropathological changes, such as deposition of amyloid plaques and neurofibrillary tangles, which result in neuronal and synaptic loss, and cortical atrophy, as well as subtle cognitive deficits (Bäckman et al. 2005; DeTure and Dickson 2019). Due to the irreversible nature of AD, current possibilities are limited to delaying onset of the disease or slowing its progression. Interventions based on modifiable risk and protective factors (Imtiaz et al. 2014; Livingston et al. 2017; Xu et al. 2015) can only be successful when targeted before significant pathological changes and cognitive decline have occurred (DeKosky 2003). Cognitive decline resulting from AD pathology occurs in several domains, over a long period of time, up to over a decade before individuals meet clinical criteria for AD (Amieva et al. 2008; Chen et al. 2001). AD is a clinically heterogeneous disease, often difficult to distinguish from normal cognitive ageing in the early and preclinical stages of the disease. Episodic memory impairment is commonly reported in early AD stages. However, an important diagnostic step forward has been that it is no longer seen as the defining symptom (Lim et al. 2020), as impairment may be evident in several other domains, including executive functions, visuospatial ability, or language, in the form of reduced complexity of sentences or anomia (Galton et al. 2000). Considering the heterogeneity in presentation of the disease, the irreversible nature, as well as the increasing emphasis on characterization of clinical and preclinical stages of AD as a continuum (Jack et al. 2018), it is crucial to develop assessment tools that can identify the subtle cognitive changes early on that indicate underlying pathology before AD is clinically evident.

MCI was introduced as a transitional phase between cognitive ageing and dementia, which is characterized by some decline in one or two cognitive domains without marked functional impairment, making it a target stage for interventions. Reported rate of conversion from MCI to dementia varies widely, depending on a number of factors, including, but not limited to, subtype of MCI, level of cognitive impairment, length of follow-up, loss to follow-up, and study setting (Ward et al. 2013). Generally, an annualized conversion rate of 10% to 15% has been widely cited, with this rate being as high as 28% for the amnestic subtype (Schmidtke and Hermeneit 2008). It has, however, been challenging to detect subtle changes occurring due to pathology during this stage, to distinguish MCI from age-related cognitive decline, and to predict conversion to dementia; however, it has been suggested that combining several markers greatly increases predictive power (Devanand et al. 2008). Therefore, continued efforts are required in the detection of MCI and in predicting conversion to dementia.

### The role of discourse processing as a potentially important early marker of AD and MCI

Assessment tools that are able to detect pathology-related cognitive decline early in the course of the disease remains a challenging field looking for innovative approaches. Established neuropsychological testing includes the Mini-Mental Status Examination (MMSE) as a screening tool, verbal fluency and the Boston Naming Test (BNT) for measuring language abilities, the logical memory subscale from Weschler’s Memory Scale for measuring episodic memory, constructional praxis for measuring visuoconstructive abilities, and the Trail Making Test (TMT) to measure executive functions. Language functions are preserved for longer, and reveal rather low vulnerability during healthy ageing (Park and Reuter-Lorenz 2009). Classic cognitive testing, so far, taps into language-related functions only marginally (Cummings et al. 1988; Taler and Phillips 2008; Verma and Howard 2012; Vuorinen et al. 2000), using tasks involving word retrieval, verbal fluency, and word list memory.

Most studies have suggested impairment primarily in the lexical and semantic components of language (Emery 2000; Henry et al. 2004; Reilly et al. 2011), which is central for relating the concept to the linguistic form. In contrast, syntactic and phonological components appear to be relatively preserved, until the advanced stages of the disease, although syntactic complexity is reduced (Emery 2000; Rochon et al. 1994). These methods for studying language-related functions, however, are rather artificial as they lack any context, and have little ecological validity. There is also considerable heterogeneity in the patterns of cognitive and linguistic decline observed, and different language functions may be variably affected in different individuals (Cummings 2000), which may not always be captured by studying language functions in isolation, such as lexical access, verbal memory, or syntactic complexity.

A more holistic approach is to study language deficits in their interactions with cognitive processes. Linguistic and cognitive processes are highly interdependent, with language shaping cognitive processes—including non-verbal processes, such as visual perception or memory—and cognition, in turn, aiding higher-order linguistic processes (Gerwien and von Stutterheim 2018). Here, we focus on discourse as a highly demanding task involving interdependency of cognitive and linguistic processes. Discourse refers to written or spoken language in a social context, and according to most definitions, encompasses information distributed over more than one sentence. Despite syntactical preservation, production of discourse is impaired very early on in the course of the disease, even before the onset of other clinical symptoms, as evidenced in studies using spontaneous speech and picture description tasks (Mueller et al. 2018; Slegers et al. 2018).

Importantly, discourse processing is qualitatively different from isolated linguistic tasks or even sentence processing. It occurs simultaneously on multiple representational levels, namely, surface code, textbase, and situation model (Fletcher and Chrysler 1990; Graesser et al. 1997). The most basic and superficial level of representation is the *surface code*, which simply preserves the exact syntax and wording of the text, generally for a few seconds only. The *textbase* is a representation of the text at a semantic level, extracting and retaining meaning from the text by inferencing, but not retaining the exact details of the text. Finally, the *situation model* refers to the level of representation wherein overall meaning of the text is interpreted in the wider context of structured world knowledge. These final two levels of processing require an interaction between cognitive and linguistic processes, as it involves abstraction, organization of information, contextual embedding, accessing appropriate schemata, incorporating relevant knowledge structures, perspective taking, and inferencing (Sparks 2012; Thorndyke 1976). Macrostructural organization is an essential property at the textbase level as well as at the level of the situational model, relevant for establishing global coherence (Kintsch 1988; Kintsch and Rawson 2005). *Macrostructural processing* is a form of higher-level language processing, which involves the representation of the global meaning of discourse in the form of the topic, theme, or gist, as opposed to *microstructural processing*, which is a very local form of processing, involving linguistic structure at the phrasal or sentence level, and meaning of words (Van Dijk 2019).

Considering the complexity of the processing involved at the macrostructural level, it may be particularly susceptible to decline early in the course of AD development. This has in fact been observed in studies using a discourse production paradigm, wherein, macrolinguistic features of discourse production were the most susceptible to decline in the early and prodromal stages of AD (Brandão et al. 2013; Pistono et al. 2019). The patterns of deficits observed in micro- and macrostructural processing have been shown to have utility in distinguishing clinical populations (Ulatowska et al. 1999). They were able to successfully distinguish individuals with MCI due to AD from those with MCI due to non-AD pathologies (Mazzon et al. 2019). Further, studies indicate that macrostructural level comprehension remains intact in normal cognitive ageing; in fact, older adults rely increasingly on this form of processing, in order to compensate for decline in detail-level memory (Radvansky and Dijkstra 2007; Ulatowska et al. 1998). Hence, emerging research targeting discourse comprehension at a macrostructural level may have the potential to add to the ongoing discussion on early markers of pathology, and in distinguishing normal cognitive ageing from AD pathology-related decline. Therefore, a systematic account of the available evidence in this area is needed.

### Goals of review

The overarching goal of this review is to evaluate currently available research measuring macrostructural discourse comprehension in the course of AD, and to assess the potential of a discourse comprehension paradigm as a novel approach in neuropsychological testing, in seeing what it may add to current testing practices. The review focuses on studies with individuals with late-onset early stage AD (mild or early moderate) and individuals with MCI, in comparison with cognitively healthy older adults. Subgoals of our review are, first, to systematize and characterize the measures of macrostructural discourse comprehension, applied in relevant studies. Second, we evaluated the associations between measures of discourse comprehension and cognitive and neuropsychological test measures that are commonly in use in clinical settings.

## Method

### Search strategy

A literature review was performed using the methods specified in the Preferred Reporting Items for Systematic Reviews and Meta-Analyses (PRISMA; see S1). We searched PubMed, Web of Science, and PsycINFO for original, peer-reviewed research articles published in English, using combinations of the following search terms: Alzheimer’s disease, mild cognitive impairment, discourse, global coherence, macrolinguistic, connected speech, connected language, narrative speech, narrative comprehension.^1^ We placed no restrictions based on date of publication of a study (for detailed search strings, see S2).

The searches were completed on January 20, 2020. Two researchers (EK and SC) screened the title and abstract of articles. When abstracts did not contain enough information to determine inclusion or exclusion, the full text of the article was obtained and read. Additionally, the references of included studies were screened to identify any other studies that may meet the inclusion criteria. Any conflicts between the two reviewers were discussed and resolved.

### Study selection

For a study to be included in the review, the following criteria had to be met: (1) the study included a group of participants who had a formal diagnosis of Alzheimer’s disease or Mild Cognitive Impairment, using well-established criteria; (2) the study included a healthy control group for comparison; (3) mean age of the healthy group was ≥ 60 years, or population was age-matched to the patient group; (4) study consisted of a text followed by outcomes measuring overall comprehension of text; (v) study was published in English in a peer-reviewed journal. The criteria for exclusion were as following: (1) Studies with other types of dementia population; (2) studies measuring verbatim recall of discourse texts or only memory for details within the text; (3) studies measuring spontaneous or picture-elicited discourse production; (4) case studies. No restrictions were placed on the type of study design.

### Data extraction

The reviewers (EK and SC) extracted the following data from the articles that were finally included in synthesis: first author’s last name, year of publication, participant groups, number of participants, age, country in which study was conducted, language of study, stage of Alzheimer’s/MCI, diagnostic criteria used, variables controlled for, task, outcome measures.

### Quality assessment

The Standard Quality Assessment Criteria for Evaluating Primary Research Papers: Quality Scoring for Quantitative Studies or ‘QualSyst’ (Kmet et al. 2004) was used to assess and rate the quality of the studies that were finally included in the analysis. The assessment originally contained a total of fourteen questions, of which, two questions concerning ‘intervention’ were eliminated, as the review did not include intervention studies. There were three possible scores for each question. A score of ‘2’ indicated the study fulfilled the criteria fully, a score of ‘1’ indicated a partial fulfilment of the criteria, and when criteria was not fulfilled, a score of ‘0’ was given. The score obtained for each study was then divided by the total possible score (24 points), giving a score between 0 and 1. Two raters (EK and SC) scored the studies independently, and a good inter-rater agreement was observed (ICC = 0.87). Any discrepancies in scoring between the two raters were discussed until consensus was reached. The quality score for the individual studies is presented in Table 1. All studies were deemed to be of a fairly good quality (≥ 0.75).

**Table 1:** Characteristics of included studies

References	Population (*N*)	Mean age	Language; country	Stage	Linguistic task	Discourse comprehension measures	Variables controlled for	Diagnostic criteria used (staging)	Quality assessment rating
Almor et al. (2001)	AD (10), NC (10)	AD = 82, NC = 78	English; USA	Mild to moderate	Reading aloud visual target words continuing from auditory stimuli	Naming latencies	(Age, education)*	NINCDS-ADRDA^a^ (MMSE^b^)	0.77
Chapman et al. (1998)	AD (10), Fluent Aphasia (10), NC (10)	AD = 65, FA = 65, NC = 65	English; USA	Mild to early moderate	Summarizing fables	Gist, lesson of stories, main idea	Age, education, sex	NINCDS-ADRDA (MMSE)	0.75
Chapman et al. (2002)	AD (24), MCI (20), NC (25)	AD = 72.4, MCI = 72.7, NC = 76.1	English; USA	Mild	Summarizing biographical narratives	Summary, main idea, lesson of stories	Age, sex	NINCDS-ADRDA (MMSE, CDR^c^); Petersen et al. 1999	0.83
Chapman et al. (2006)	AD (12), Young OA (12), Old OA (12)	AD = 71.6, YOA = 72.2, OOA = 85.8	English; USA	Mild	Summarizing a narrative; Logical Memory Subtest of WMS-III	Transformed gist, main idea	Education, sex, depression	NINCDS-ADRDA	0.83
Creamer and Schmitter-Edgecombe (2010)	AD (20), NC (20)	AD = 77.2, NC = 76.7	English; USA	Mild	Think-aloud while reading stories	Proportion of inferential clauses	Age, education, sex	NINCDS-ADRDA (CDR)	0.96
Drummond et al. (2019)	AD (14), aMCI (31), NC (39)	AD = 75.3, aMCI = 72.2, NC = 71.8	Portuguese; Brazil	Mild	Summarizing narrative story	Main ideas, comprehension questions, inferential lesson	Age, education, sex	DSM-5^d^; Winblad et al., 2004	0.88
Schmitter-Edgecombe and Creamer (2010)	aMCI (23), NC (23)	MCI = 70.8, NC = 70.6	English; USA	MCI	Think-aloud while reading stories	Proportion of inferential clauses	Age, education, sex	Petersen et al., 200l; CDR	0.96
Welland et al. (2002)	EDAT (8), MDAT (8), NC (8)	EDAT = 78, MDAT = 76.7, NC = 72.2	English; Canada	Mild and moderate	Answering yes/no comprehension questions about narratives	Implied main ideas and implied details questions	Age, education, IQ	NINCDS-ADRDA (MMSE)	0.83

^a^National Institute of Neurological and Communicative Diseases-Alzheimer’s Disease and Related Disorders Association (McKhann et al. 1984)

^b^Mini-Mental State Examination (Folstein et al. 1975)

^c^Clinical Dementia Rating (Hughes et al. 1982)

^d^The Diagnostic and Statistical Manual of Mental Disorders, Fifth Edition

*Entered as covariates

## Results

### Search results and study characteristics

**Fig. 1:**
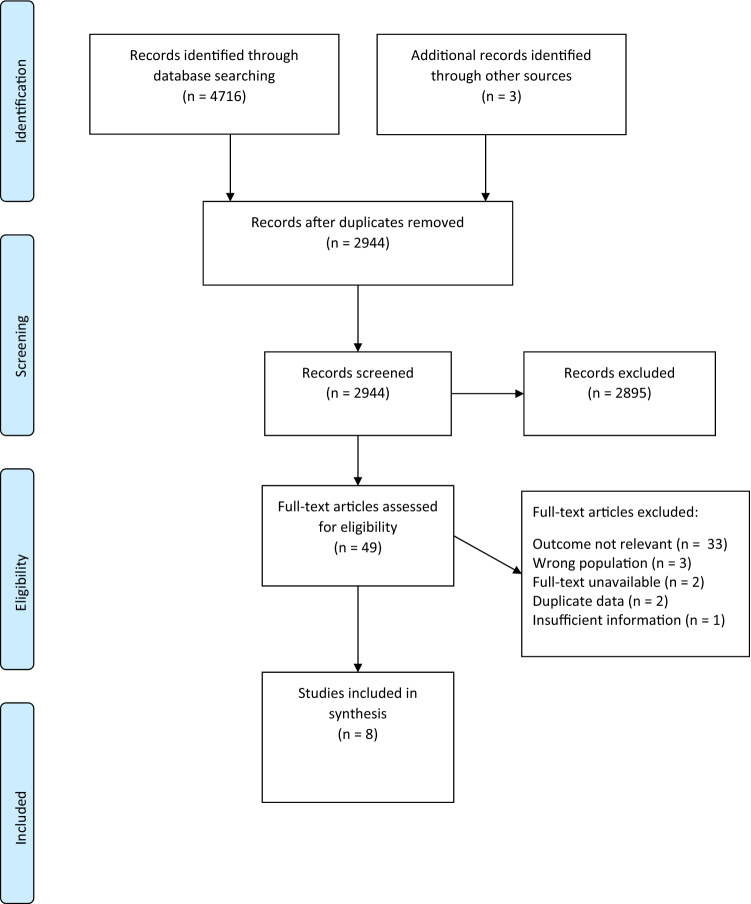
Flowchart of literature search and study selection process

The search yielded a total of 4716 articles combined from PubMed (1954–2020), Web of Science (1934–2020), and PsycINFO/EBSCO (1934–2020). After removing duplicates 2941 articles remained, for which title and abstract were screened. Additionally, references of included articles were screened, and three additional articles, which met the inclusion criteria, were identified (Chapman et al. 2006; Graville and Rau 1991; MacDonald et al. 2001), making it a total of 2944 articles that were screened for eligibility. Of these, 2895 articles were excluded as they did not pertain to the topic or did not meet inclusion criteria. Full-text screening was conducted, and inclusion and exclusion criteria were applied for the remaining 49 articles. Of these, 41 articles were excluded, with a good inter-rater agreement (*κ* = 0.81). The reasons for exclusion are highlighted in Fig. 1. The most common reason for exclusion was ‘Outcome not relevant’ with most studies being excluded as they investigated spontaneous or picture-elicited discourse production or verbatim recall of text. Finally, a total of eight articles were included in the review, which aimed to measure discourse comprehension at a macrolevel, in adults with Alzheimer’s disease or MCI.

An overview of the study characteristics is presented in Table 1. All the studies were cross-sectional, in which AD and/or MCI groups were compared to cognitively healthy older adults. Seven of the eight studies were conducted with native English-speakers, with six of them being conducted in USA, and one in Canada. One study was conducted in Brazil, with a native Brazilian Portuguese-speaking population. The studies were published between the years 1998 and 2019. One study included two groups of healthy older adults, classified as ‘young-older adults’ (65–80 years) and ‘old-older adults’ (> 80 years) (Chapman et al. 2006), and one study (Welland et al. 2002) included two AD groups—early stage (EDAT) and moderate stage (MDAT). The total sample sizes ranged from 20 to 84 participants, with their mean ages ranging from 65 to 86. All studies controlled for age, and all but one (Chapman et al. 2002) controlled for education, wherein the different groups were either matched on these variables or the variables were entered as covariates during analysis. Apart from this, six studies also controlled for sex (Chapman et al. 1998, 2002, 2006; Creamer and Schmitter-Edgecombe 2010; Drummond et al. 2019; Schmitter-Edgecombe and Creamer 2010), one study controlled for depression(Chapman et al. 2006), and one study controlled for IQ (Welland et al. 2002). All studies determined cognitive status of the healthy control group using at least one or a combination of several of the following measures—MMSE, self-report, Clinical Dementia Rating (CDR), Global Deterioration Scale (GDS).

Only one study (Drummond et al. 2019) used a test from a standardized battery (MAC battery) (Fonseca et al. 2008), and one (Welland et al. 2002) used a modified form of the Discourse Comprehension Test (DCT) battery (Brookshire and Nicholas 1993) to measure discourse comprehension. In other studies, an experimental task was used to measure discourse comprehension, wherein participants were presented with a series of short texts, usually narrative stories. This was generally followed by a variety of tasks designed to test participants’ comprehension of the texts. This involved giving a short summary of the story, stating the lesson or intended main idea of the story, answering true/false questions about the story, a think-aloud paradigm while reading, or reading out loud the last word in the story, which was either congruent or incongruent with previous text. With one exception (Welland et al. 2002), the studies did not report independently on hearing and visual/reading abilities of participants. However, they generally included practice trials before the start of the study to ensure participants understood the task, and were able to perform it successfully. Almost all of the included studies looked at performance of participants on one or more neuropsychological tests (for example, subtests of Boston Diagnostic Aphasia Examination) to ensure that participants were able to follow instructions, in order to be able to perform the task. The outcome measures varied across studies, with some studies measuring the proportion of inferential and non-inferential clauses produced (Creamer and Schmitter-Edgecombe 2010; Schmitter-Edgecombe and Creamer 2010), one study measuring naming latencies for congruent and incongruent pronouns (Almor et al. 2001), and others measuring gist-level retelling in the form of summary, lesson, main ideas (Chapman et al. 1998, 2002, 2006; Welland et al. 2002). Due to this heterogeneity in tasks and reported outcome measures, a meta-analysis was not performed.

### Diagnostic criteria

One study (Drummond et al. 2019) used the Diagnostic and Statistical Manual of Mental Disorders: Fifth Edition (DSM-5) criteria for Major Neurocognitive Disorder due to Alzheimer’s Disease (Sachdev et al. 2014), for diagnosis of AD. All other studies used the National Institute of Neurological and Communicative Disorders and Stroke and the Alzheimer’s Disease and Related Disorders Association (NINCDS-ADRDA) criteria (McKhann et al. 1984). In all studies, a diagnosis of ‘probable AD’ was applied, wherein individuals are diagnosed based on clinical and neuropsychological evidence without histopathologic confirmation. As these were cross-sectional studies, they could not follow-up to confirm AD via autopsy. Additionally, all, but one, studies were conducted prior to 2011, when the NINCDS-ADRDA criteria were first revised to the National Institute on Aging-Alzheimer’s Association (NIA-AA), to include biomarker evidence in the diagnosis of AD (McKhann et al. 2011). The DSM-5 criteria, which was used in the study by Drummond et al. (2019), does not yet include biomarker evidence in diagnosis of Major Neurocognitive Disorder due to AD. The major difference between the NINCDS-ADRDA and the DSM-5 criteria is that presence of memory impairment is not required for diagnosis in DSM-5; rather, impairment in any two cognitive domains is acceptable. This shows a general trend towards moving away from memory impairment, as is seen in the NIA-AA 2011 criteria too, which was a revision of the NINCDS-ADRDA criteria. For determining the stage of the AD (mild, moderate, severe), studies used either MMSE or CDR scale (Folstein et al. 1975; Hughes et al. 1982). These two scales have been shown to have good agreement for the stages of AD that have been investigated in included studies (Perneczky et al. 2006). Overall, although two different criteria were used for the diagnosis of AD, the criteria were comparable enough that a qualitative synthesis of studies was possible.

For a diagnosis of MCI, one study (Chapman et al. 2002) used the criteria by Petersen et al. (1999); another study (Schmitter-Edgecombe and Creamer 2010) applied the criteria by Petersen et al. (2001). The studies also ruled out other possible causes of cognitive impairment (such as stroke or other neurological or psychological causes) via a series of tests. As with the diagnostic criteria for AD, the criteria for MCI too evolved to shift focus away from memory complaints, towards a more wholesome approach to include all cognitive domains. While the Petersen et al. (1999) criteria required a subjective memory complaint, the subsequent revised criteria from 2001 onwards allowed for complaints in any cognitive domain. Instead, the Petersen et al. (2001) criteria focused on classifying MCI into several subtypes (e.g. amnestic MCI, multi-domain MCI), depending on the cognitive domain(s) in which deficits were observed. Accordingly, studies included in the review that were conducted after the Petersen et al. (2001) criteria were established, have included population specifically with a diagnosis of amnestic MCI (aMCI). Finally, one study (Drummond et al. 2019) applied the Winblad et al. (2004) criteria, which was a revision of the Petersen et al. (2001) criteria. This revision acknowledges that there may be multiple aetiologies for each subtype of MCI, and modifies the stipulation concerning normal daily functioning in previous criteria, to allow for subtle impairment in complex functions. Although different evolving diagnostic criteria have been used in the included studies, the different criteria are not sufficiently different enough so as to affect a qualitative synthesis of these studies.

### Measures of discourse comprehension

Due to a lack of standardized tests for measuring discourse comprehension, there was considerable variability in the method used to evaluate comprehension, and consequently in the type of outcome measures used. Most measures used some form of language production to measure comprehension. This implies a general problem which poses a dilemma for comprehension studies in other contexts as well (e.g. language acquisition, pedagogy). We know from studies on language production that patients with AD have deficits in accessing lexical units, though deficits at the morphological and syntactical level are less pronounced. These deficits could affect the validity of the measures for language comprehension.

Relevant outcome measures used in each study were identified. Several of the identified outcome measures were used in multiple studies, and these were grouped together. The names of the outcome measures were derived from the outcomes used in the included studies. However, the terms for certain measures were used interchangeably in the different studies. Therefore, to summarize the results from different studies, the measures were categorized according to the definitions or descriptions of the measure presented in the studies, rather than the terms used. Accordingly, the measures were grouped into the six variables described below. The results for each measure are summarized in Table 2.

**Table 2:** Comparison of group performance on discourse comprehension measures

References	Naming latencies	Summary	Lesson/message	Main idea	Inferential clauses	Comprehension questions
Almor et al. (2001)	AD < NC***	–	–	–	–	–
Chapman et al. (1998)	–	AD < NC^†^	AD < NC^†^	AD < NC^†^	–	AD < NC^†^
Chapman et al. (2002)	–	AD = MCI < NC**	AD < MCI < NC***	AD < MCI < NC***	–	–
Chapman et al. (2006)	–	AD < OOA*** AD < YOA*** OOA < YOA*	AD < OOA*** AD < YOA*** OOA = YOA	AD < OOA** AD < YOA** OOA = YOA	–	–
Creamer and Schmitter-Edgecombe (2010)	–	–	–	–	AD < NC*	AD < NC***
Drummond et al. (2019)	–	AD < MCI < NC*	AD = MCI < NC*	–	–	AD < MCI < NC*
Schmitter-Edgecombe and Creamer (2010)	–	–	–	–	MCI < NC**	MCI < NC*
Welland et al. (2002)	–	–	–	–	–	MDAT = EDAT < NC**

*p ≤ .05, **p ≤ .01, ***p ≤ .001

^†^*p* value not reported

#### Naming latencies

Naming latency was used as an outcome in only one of the studies (Almor et al. 2001). In this study, participants were presented with a short text in an auditory format, in which two entities (antecedents) were introduced in the first sentence. The final sentence referred back to these entities, wherein it mentioned one of the entities and was left incomplete before the other entity is mentioned. Finally the target pronoun was presented visually, which was either congruent with the incomplete sentence or incongruent, based on the singularity or plurality of the antecedent and the pronoun. Participants were to read aloud the pronoun, and their response time was measured. Ideally, when the pronoun is incongruent to the antecedent, response time should be longer compared to when it is congruent, as it would be more difficult to integrate an incongruent word into the passage, indicating adequate processing of cohesive devices. This effect would, however, only be seen if individuals are able to integrate different information units within a macrostructure, indicating the ability to establish coherence relations. Slower reaction times for incongruent trials were seen in healthy older adults, as well as the group with AD. However, the size of the effect was much smaller in the AD group compared to the healthy older adults, meaning that the difference in the reaction times to congruent vs incongruent trials was much higher in the controls than in the AD population, as was expected. This shows that AD patients were less sensitive to incongruent pronouns, indicating a problem in integrating and connecting the presented information.

#### Summary

In four studies (Chapman et al. 2006, 1998, 2002; Drummond et al. 2019), participants were presented with a short story. Following this, participants were asked to retell the story or give a summary in their own words which involved focusing on important units of information that are required for an overall understanding of the story, and omitting unnecessary details. Participants’ performance was scored according to the number of main informational and/or thematic units produced. This measure can be taken to illustrate in how far language production was taken as a measure for comprehension. The linguistic output was not analysed with respect to relevant features of language production (time course, lexical choice, or number of words per sentence), but only at the level of meaning in relation to the stimulus text. AD groups produced fewer synthesized meaningful units of information compared to cognitively healthy adults in all four studies, including the old-older adults. In both studies with MCI population (Chapman et al. 2002; Drummond et al. 2019), the MCI group performed significantly worse than the healthy older adults. Between the AD and MCI groups, AD group scored significantly lower than the MCI group in one study (Drummond et al. 2019); however, the performance of the two groups was comparable in another study (Chapman et al. 2002). Additionally, there was a small but significant difference in the performance of old-older adults compared to young-older adults. This was the only measure for which such a difference was observed.

#### Lesson/message

Another probe following the presentation of a short story, employed in four studies (Chapman et al. 2006, 1998, 2002; Drummond et al. 2019), was the lesson or message probe, wherein participants were to formulate a lesson or a title that could be inferred from the story. AD and MCI patients scored significantly lower than healthy adults, focusing on unimportant details from the story rather than an overall lesson. Additionally, the AD group performed significantly worse than old-older adults. When performances of MCI and AD groups were compared, the results were mixed, wherein one study (Drummond et al. 2019) reported no significant difference in their performance, whereas another study (Chapman et al. 2002) reported that the AD group scored significantly lower than the MCI group. This measure required maximum inferential processing, as participants need to be able to synthesize a large amount of information, condense it, and make interpretations about what message it carries.

#### Main idea

This probe, also administered following a short story in three of the studies (Chapman et al. 2006, 1998, 2002), measured the ability of participants to summarize the story in one sentence i.e. the primary concept of the story, which required substantial condensation of information and abstraction into one generalized idea. Both AD and MCI groups performed significantly worse than the control group. Furthermore, a significant difference was observed between the performance of AD and MCI groups, with the AD group scoring lower than the MCI group. AD and MCI patients were generally prone to giving more unimportant information or details rather than summarizing statements, although individuals’ responses varied to some extent. Additionally, as was also observed for previous measures, the AD group’s performance was significantly worse compared to the old-older adults.

#### Inferential clauses

Two studies used a think-aloud procedure (Creamer and Schmitter-Edgecombe 2010; Schmitter-Edgecombe and Creamer 2010), wherein participants were given a short narrative text to read, and were asked to vocalize their thoughts about the story simultaneously while reading the narrative text. Every utterance of participants was classified either as an ‘inferential clause’ or a ‘non-inferential clause’, by two assessors, one of whom was blinded to the diagnostic status. The classification system used by Trabasso and Magliano (1996) was employed, wherein, statements that were either explanations, predictions, or formed associations, were categorized as ‘inferential’, and other statements (e.g. repetitions or paraphrases) were classified as ‘non-inferential’. Although, overall, all groups uttered more inferential clauses compared to non-inferential, both AD and MCI groups uttered significantly fewer inferential clauses compared to cognitively healthy adults.

#### Comprehension questions

One included study (Welland et al. 2002) used Yes/No questions as the only outcome to measure comprehension following story narration. The format used in this study was adapted from the standardized discourse comprehension test developed by Brookshire and Nicholas (1993). The questions were categorized based on the level of detail—main idea and details, and the type of information—implied or stated. Both patient groups—EDAT and MDAT—performed significantly worse on all types of questions, compared to the healthy group, but the performance of the two patient groups did not differ from one another on any measure. All groups generally performed better on ‘main idea’ questions compared to ‘details’, and on ‘stated’ information compared to ‘implied’. Three other studies (Creamer and Schmitter-Edgecombe 2010; Drummond et al. 2019; Schmitter-Edgecombe and Creamer 2010) included comprehension questions following the other retelling and ‘think-aloud’ tasks, to test for comprehension of the narrative passage. In two studies, half of the True/False questions were based on information that needed to be inferred from the text and half of the questions were based on facts that were explicitly stated in the text. AD and MCI groups answered fewer questions correctly, overall, compared to controls, in all studies. However, when performance on inferential questions was examined specifically, in the two studies that made this distinction, AD and MCI groups did not differ significantly from controls. Therefore, in these studies, this measure was relatively less informative, as the nature of the questions (True/False) poses two problems. First, there is a 50% chance of answering the question correctly, irrespective of how well one may or may not have understood the narrative. This can be observed in the AD group’s performance, which was in fact at chance level. Second, there may be possible ceiling effects in the healthy adults group’s performance, as can be observed in the high means across all the studies. It is also possible that performance on this task was made easier by reliance on recognition memory, rather than recall. Therefore, this method may not be optimal in terms of appropriateness and complexity in investigating the current question.

Overall, a deficit in discourse comprehension in individuals with AD and MCI was consistently observed across all studies, pointing to a robust effect. These result show that, with the exception of one measure, discourse comprehension measures are able to reliably distinguish early stage AD and MCI patients from cognitively healthy older adults.

### Association between discourse comprehension measures and cognitive measures

In addition to examining the discourse comprehension differences between AD, MCI, and cognitively healthy older adults, the review also aimed to examine whether performance on the discourse comprehension task correlated with performance on commonly used neuropsychological tests. The purpose of this was twofold: the first was to examine which cognitive processes, if any, are able to predict performance on a discourse comprehension task, giving an indication of the underlying mechanisms involved; the second was to determine whether discourse comprehension tasks are able to tap into processes beyond what traditionally used neuropsychological tests measure. Studies used tests such as RAVLT, WAIS-III, listening span, D-KEFS, MMSE to measure verbal memory, working memory, executive functions. However, all these measures were not consistently used across all included studies. Therefore, it was somewhat challenging to draw robust conclusions about their association with discourse comprehension. For measures that were employed in multiple studies, the results were mostly mixed. When the association between MMSE scores and performance on the experimental task were examined, one study (Chapman et al. 2002) found a significant correlation (*r* = 0.65), whereas another study (Almor et al. 2001) found only a marginally significant correlation between the two measures, which disappeared when working memory was accounted for. In another study (Welland et al. 2002), MMSE scores did not significantly predict discourse comprehension when episodic memory or working memory were added to the regression model. Similarly, working memory measures were associated significantly (*r* = 0.64, *r* = -0.83) with discourse comprehension in two studies (Almor et al. 2001; Welland et al. 2002), but two other studies (Creamer and Schmitter-Edgecombe 2010; Schmitter-Edgecombe and Creamer 2010) found no association. It is important to note that different studies used different tests to measure working memory (e.g. listening span, WAIS-III, digit span). These varying results may be due to heterogeneity in the different experimental tasks and tests used in different studies. However, both studies that included a verbal memory measure (RAVLT) found a significant, albeit moderate (*r* = 0.50 to *r* = 0.64) correlation with discourse comprehension measures. Only one study (Welland et al. 2002) reported a positive association with episodic memory (*r* = 0.91*)*. Additionally, one study (Creamer and Schmitter-Edgecombe 2010) found significant correlations with TMT-A (*r* = 0.58) and D-KEFS (*r* = 0.62), measuring attention and executive functions, respectively. The study also looked at several other tests of attention and executive functions, as well as tests of language, but none of these showed association with macrostructural measures of discourse comprehension. The moderate correlation with verbal memory, and the moderate or non-significant correlations with other measures indicate that discourse comprehension tasks tap into additional processes that are not assessed by neuropsychological tests used routinely in the clinical diagnosis of AD. This warrants investigation of discourse comprehension tasks as a possibly more comprehensive assessment tool.

## Discussion

The purpose of this review was to synthesize results of studies investigating whether individuals with mild AD or MCI experience significant deficits in macrostructural discourse comprehension, in comparison with cognitively healthy older adults. In the included studies, participants were presented with short narratives, which were accompanied either by a think-aloud procedure, or were followed by a retelling of the story in short, along with questions which measured comprehension of the story. Six measures were identified from these studies—naming latencies, global synopsis, lesson, main idea, inferential clauses, and comprehension questions. Despite some variations in the methods and outcome measures across the eight studies included in the review, significant deficits in macrostructural discourse comprehension were observed in AD and MCI groups across all, but one, measures in all studies, in comparison with cognitively healthy older adults. These findings also receive additional support from results of neuroimaging and biomarkers employed in the study by Drummond et al. (2019), where they observed that performance on the discourse task was associated with the degree of neurodegeneration observed, in terms of reduced white matter integrity and neuronal loss. Although the number of studies in this review was limited, we observed a very consistent pattern of findings across the studies, indicating a rather robust effect.

The groups with AD performed significantly worse than healthy older adults on five of six measures, with one measure (comprehension questions) showing mixed results. Moreover, individuals with MCI similarly displayed significant deficits in performance when compared to the healthy groups. In studies that included both, AD and MCI groups, a direct comparison of their performance showed mixed results. On the measure of ‘main idea’, MCI group outperformed the AD group. However, for the ‘lesson’ measure, performance of the two groups was comparable in one study, whereas AD group performed worse than the MCI group in another study. Similarly, for the ‘summary’ measure, AD group performed worse than MCI group in one study, whereas their performance was comparable to the MCI group in another study. Most notably, however, one study compared performance of the AD group with the ‘old-older adults’ group (> 80 years), and found that the AD group’s performance was significantly worse on all three outcome measures included in the study. This is noteworthy, as the mean age of the ‘old-older adults’ group was significantly higher than that of the AD group. Although compared to younger adults, macrolevel comprehension shows some decline in older adults (Cohen 1979), over time it stabilizes, and is seen to be fairly preserved in the old-old, even though memory for details is generally seen to deteriorate (Radvansky and Dijkstra 2007; Ulatowska et al. 1998).

In addition to the discourse comprehension task, the studies also included some commonly used standardized cognitive and neuropsychological tests. The only measure for which an association was observed across the limited number of studies that employed it, was verbal memory, which was measured using RAVLT. A deficit in verbal memory measures has also been observed in the preclinical stage of the disease, in earlier studies (Bondi et al. 1994; Howieson et al. 1997). Even so, the strength of the correlation was moderate. It is noteworthy that all studies (Creamer and Schmitter-Edgecombe 2010; Drummond et al. 2019; Schmitter-Edgecombe and Creamer 2010) that employed commonly used verbal tasks—verbal fluency and BNT—did not find a significant association with macrostructural discourse comprehension measures, even though the measures use some form of language production, and previous discourse production studies have reported word-finding difficulties (Slegers et al. 2018). Only one study reported on correlations with episodic memory. Although the correlation was strong, the measure for which the correlation was reported was ‘Yes/No’ comprehension questions. It would be of interest to see whether there is a correlation between episodic memory performance and more complex measures such as summarizing or giving the main idea of the text. For working memory measures and MMSE, the associations produced mixed results; and when a significant association was observed, it was a moderate association. While the inconsistencies in associations may be in part due to the varying methodologies and tests used in different studies, the strength of the associations do indicate that a discourse comprehension task measures constructs beyond what classic neuropsychological tests are able to measure.

These findings highlight the need to go beyond classic cognitive and linguistic tasks (e.g. verbal fluency, confrontation naming), for a more comprehensive approach, in the neuropsychological assessment of MCI and AD. A discourse comprehension task is more representative of everyday communication and thus gives a more well-rounded picture of cognitive and linguistic deficits, over tasks measuring isolated linguistic functions. The complexity of such an assessment paradigm also means that it is perhaps a more sensitive indicator of AD pathology in the preclinical stage, although that remains to be seen, and should be an avenue for future research. Additionally, breakdown of communication is a major issue in the latter stages of AD, and is a moderating variable in determining functional independence of individuals. A discourse comprehension-based assessment tool may help track the level of functional impairment as disease progresses, and serve as a tool for targeting interventions to maintain communication ability.

The findings of this review are also notable considering that syntax and phonology are preserved in production of language during the early or even early moderate stage of AD (Kavé and Levy 2003). Evidence from studies examining spontaneous or picture-elicited discourse production shows a similar pattern of breakdown, wherein participants produce syntactically and phonologically sound sentences. However, the discourse produced was severely lacking in information content, coherence, and cohesion (Chenery and Murdoch 1994; Laine et al. 1998; Toledo et al. 2018), critical macrolinguistic features of discourse. The preservation of syntactic structure in production indicates that language processing abilities are preserved at a local, sentence-based level. Tracking information and establishing links across sentences are tasks in which the deficits show. This suggests that the comprehension deficits seen in AD patients are also more reflective of impairment in cognitive functioning, and consequently in areas where language and cognition interact. Therefore, there is a need to go beyond testing paradigms that study linguistic and cognitive functions independently of the other.

While there has been considerable research looking at patterns of language impairment in AD, this research has been conducted primarily using laboratory tasks such as word lists, confrontational naming, and word definitions, which measure individual language functions in isolation from others. These same testing paradigms are then used for assessment of linguistic functions in clinical practice too. Such paradigms do not transfer to situations that people encounter in everyday life, lacking ecological validity. They give us limited insight into individual language functions, such as lexical access or semantic fluency, but no insight into the multi-level processing of language use. Therefore, the impairments seen in AD patients during communication are often attributed to lexico-semantic deficits (Price et al. 1993; Reilly et al. 2011). Considering that deficits were observed on macrostructural measures of comprehension, as shown in this review, we cannot attribute communication deficits in AD to simply one linguistic component.

Everyday communication occurs in the form of situated discourse, which involves more than simple retention and retrieval of word lists in a contextual vacuum. Production and comprehension of discourse necessitates higher-order information processing, which requires interaction of linguistic and cognitive processes. This includes integration of context, accessing the appropriate schema, understanding goals and intentions of the communicative counterpart, merging of information in the text and semantic knowledge, generating inferences, or simply deletion of superfluous or redundant details (Kintsch and Van Dijk 1978). Such an assessment paradigm that is rooted in the practicalities of everyday interactions and experiences, provides a holistic approach in understanding cognitive and linguistic deficits in AD, offering a new dimension to neuropsychological testing practices and interventions. Previous studies with individuals with Traumatic Brain Injury (TBI) have also reported macrolevel abstraction and comprehension deficits in this population (Vas et al. 2015). They showed tasks employing macrolevel processing to have high sensitivity and specificity in TBI due to the complexity of processing required (Vas et al. 2016). With processing occurring simultaneously on multiple levels, any number of variables could be manipulated in order to pinpoint the areas where interventions should be targeted. Emerging evidence indicates that cognitive training in MCI patients that targets macrolevel processing not only benefits abstraction ability, but also extends to other general cognitive functions like attention and executive functions (Chapman and Mudar 2014; Das et al. 2019), and is also linked to brain changes (Mudar et al. 2019).

Finally, as identified from previous studies, executive functions, episodic memory, semantic memory, and working memory play important roles in discourse comprehension (Calvo 2001; Cohen 1979; Daneman and Merikle 1996; Just and Carpenter 1992). It is possible that deficits seen in macrostructural comprehension may be in part due to impairment in any one of these, or possibly even multiple processes. There is evidence already that these processes are impaired in AD (Belleville et al. 2007; Huntley and Howard 2010). And, although, possibly all of these processes may be implicated in the deficits observed, which of these play a greater role remains to be seen.

### Limitations

There are several limitations to this review. First, the review was limited to studies published in English, which may also somewhat limit the countries where the included studies were primarily conducted. Another major limitation is the low number and types of studies, due to the limited literature existing in this area of research, reflecting the low emphasis on studying interaction of linguistic and cognitive processes in AD.

A further limitation is the heterogeneity of the tasks used in the studies. Due to a lack of standardized tests measuring discourse comprehension, the studies varied in the procedure and measures implemented. As a result, a meta-analysis was not conducted, which somewhat limits synthesis of the results. Further, there is a lack of consistency in the neuropsychological tests applied in the different studies. Therefore, it was difficult to draw robust conclusions about the association between cognitive abilities and discourse comprehension, and which abilities contribute to the deficits observed. Future studies should closely examine these associations.

A major limitation of the literature is the lack of longitudinal studies. Although the review placed no restriction on the type of study design, none of the studies followed-up with participants to track their trajectory. This would be especially crucial with MCI patients, as it is presently difficult to predict conversion to dementia. Another possible limitation in studying macrostructural comprehension lies in the tediousness of the procedure for analysing discourse. The linguistic expertise required to meet the standards in this field is often not available. However, there have been efforts in the past few years to simplify the procedure and to develop standardized measures for discourse analysis (Dalton et al. 2019). Additionally, recent advances in computational linguistics are promising, with major components of the analyses being automatized, making the process less time consuming and less error-prone (Aluisio et al. 2016; Clarke et al. 2020).

Finally, as addressed previously, most of the included studies employ tasks which use some form of language production to measure comprehension. This is disadvantageous to individuals whose comprehension ability may be unaffected, but who may be experiencing deficits in production of language. This issue can be resolved using tasks which do not involve production, or even entirely non-verbal tasks that measure macrostructural processing by using other cognitive domains such as in visual world paradigms.

### Future directions

This review highlights the potential of discourse comprehension measures as such a novel, comprehensive approach towards neuropsychological assessment that is able to capture cognitive and linguistic variables at multiple levels—microstructural, macrostructural, pragmatic, grammatical. Given the consistent findings despite some methodological variations across studies, its sensitivity during the early and preclinical stage of AD (MCI), and its advantage over classic cognitive tests, it warrants further research with more linguistically and culturally diverse populations, and an attempt to establish a standardized format for the test, with the aim of early detection of pathology.

In one study, it was observed that individuals with AD that scored in the normal range on MMSE showed difficulties in discourse comprehension. Additionally, two studies reported that MMSE scores were not associated with performance on discourse comprehension measures. This indicates that task paradigms such as those used in the studies included in this review may be more sensitive in the early stage of the disease. This is also evident in the performance of the MCI group, which was significantly worse than the healthy group, in all the studies that included these patients. Such paradigms for assessment may also be advantageous when considering individuals with a high cognitive reserve (CR), who take longer to show clinical indication of AD, when tested using classic neuropsychological assessment tools. It has, however, been suggested that using more complex and challenging tasks may be better able to detect the presence of pathology in this challenging group (Stern 2012, 2013).

In recent years, a number of reliable biomarkers of AD have been identified (Khoury and Ghossoub 2019). Consequently, this has opened up the possibility of detecting AD in its preclinical stage, when individuals show no cognitive deficits on standard neuropsychological assessments (Haldenwanger et al. 2010; Villemagne et al. 2011). The preclinical stage of AD is, however, characterized by subtle cognitive deficits. Although standard neuropsychological assessments, using simple, isolated tests of language and cognition may not able to detect AD pathology during the preclinical stage, this is not necessarily the case for more complex cognitive tasks. In some recent studies that used cognitive tasks requiring more complex processing (e.g. face name association task, memory binding task), significant deficits in performance were observed in preclinical AD population (Rentz et al. 2013; Tort-Merino et al. 2017). In the study by Drummond et al. (2019), which was included in this review, it was observed that severity of deficits on discourse task correlated with the degree of neurodegeneration, as measured through neuroimaging and CSF biomarkers, in the AD group. A combination of biomarkers and comprehensive cognitive testing has shown more promise in predicting clinical outcomes, over biomarkers alone (Bondi and Smith 2014). Future studies should aim for a translational approach to investigate discourse comprehension ability in preclinical AD population and its association with AD biomarkers, for the potential development of a robust assessment tool for the early detection of AD pathology in clinical settings, where biomarker use is uncommon.

Additionally, in studies in this review that included both MCI and AD groups, performance of the two groups was comparable on some measure, but significantly different on other measures. Upon closer examination, it was observed that just over half of the individuals with MCI displayed deficits in discourse comprehension, whereas the performance of the rest of the group was comparable to the healthy older adults. Previous research has shown that MCI patients who go on to convert to dementia show more severe impairment in some linguistic and cognitive domains, compared to those who do not  convert (Celsis 2000). Another study also showed disparate profiles of MCI patients in a text comprehension task (Chesneau et al. 2016). It is of interest to find predictors of conversion, and this approach shows preliminary promise.

Finally, it has been suggested that neuropsychological testing should move into a new direction, focusing on novel approaches, especially in populations in prodromal stages of the disease, when classic neuropsychological tests are unable to detect underlying pathology (Rentz et al. 2013). Macrostructural processing, which taps into top-down processes, seems to be a promising area for such research. A multi-dimensional approach, combining several biological and cognitive-linguistic predictors, also helps to track cognitive changes over time and our ability to predict clinical outcomes (Bondi et al. 2008). While discourse processing is one paradigm that taps into these processes, other approaches for testing comprehension at a macrostructural level, extending to non-verbal paradigms as well, are warranted to measure and understand the decline from the prodromal stage of AD to the clinical stage.

## Conclusion

Individuals with AD and MCI experience significant deficits in discourse comprehension, which are not otherwise seen in cognitively normally ageing adults, irrespective of their age. These deficits are present in the early stage of AD, and only show moderate correlation with verbal memory and working memory capacity measures, indicating that they tap into additional constructs. With the increasing emphasis on identifying and characterizing the preclinical stages of AD in order to target interventions, more studies are focusing on such novel approaches, which have shown promising results. Studying impairment in AD using tasks which require multilevel cognitive processing, integrating knowledge from different sources and modalities, could reveal deficits which do not show in less complex processes, at this stage. We conclude on the basis of the results obtained that studies which use measures that tap into top-down processes rather than studying individual linguistic and cognitive components might serve this purpose, finally leading to a diagnostic tool with clinical utility in early detection. Such an approach has utility in research and clinical settings for differential diagnosis, for predicting conversion from MCI to dementia, and also as a tool for training intervention in older adults who experience a subjective decline in cognitive functions. Longitudinal studies, beginning before clinical onset of AD, are required to determine the potential of this assessment paradigm to identify indicators of AD pathology during the preclinical stage. Additionally, further studies to increase reliability and validity of this measure, and translational studies which include neuroimaging and biomarkers, are warranted to investigate the potential of discourse comprehension assessment paradigm for these purposes.

## Supplementary Information

Below is the link to the electronic supplementary material.

Supplementary file1 (DOCX 16 kb)

Supplementary file2 (DOCX 12 kb)

## Footnotes

1 As per recommendation from one reviewer, we conducted an additional search with the search terms ‘gist’, ‘inference’, and ‘text comprehension’ in combination with ‘Alzheimer’s disease’ and ‘Mild Cognitive Impairment’ to potentially identify articles we may have missed in our original search. However, this search did not yield any new articles that met our criteria. These search results have not been added to the original search results.

## References

[CR1] Almor A, MacDonald MC, Kempler D, Andersen ES, Tyler LK (2001). Comprehension of long distance number agreement in probable Alzheimer's disease. Lang Cognit Process.

[CR2] Aluisio S, Cunha A, Toledo C, Scarton C (2016) Computational tool for automated language production analysis aimed at dementia diagnosis. In: International conference on computational processing of the Portuguese language, demonstration session

[CR3] Amieva H (2008). Prodromal Alzheimer's disease: successive emergence of the clinical symptoms. Ann Neurol.

[CR4] Bäckman L, Jones S, Berger A-K, Laukka EJ, Small BJ (2005). Cognitive impairment in preclinical Alzheimer's disease: a meta-analysis. Neuropsychology.

[CR5] Belleville S, Chertkow H, Gauthier S (2007). Working memory and control of attention in persons with Alzheimer's disease and mild cognitive impairment. Neuropsychology.

[CR6] Bondi MW, Smith GE (2014). Mild cognitive impairment: a concept and diagnostic entity in need of input from neuropsychology. J Int Neuropsychol Soc.

[CR7] Bondi MW, Monsch AU, Galasko D, Butters N, Salmon DP, Delis DC (1994). Preclinical cognitive markers of dementia of the Alzheimer type. Neuropsychology.

[CR8] Bondi MW, Jak AJ, Delano-Wood L, Jacobson MW, Delis DC, Salmon DP (2008). Neuropsychological contributions to the early identification of Alzheimer’s disease. Neuropsychol Rev.

[CR9] Brandão L, Lima TM, Parente MAdMP, Peña-Casanova J (2013). Discourse coherence and its relation with cognition in Alzheimer’s disease. Rev Psicol Pesq.

[CR10] Brookshire RH, Nicholas LE (1993) Discourse comprehension test. Communication Skill Builders

[CR11] Calvo MG (2001). Working memory and inferences: evidence from eye fixations during reading. Memory.

[CR12] Celsis P (2000). Age-related cognitive decline, mild cognitive impairment or preclinical Alzheimer's disease?. Ann Med.

[CR13] Chapman SB, Mudar RA (2014). Enhancement of cognitive and neural functions through complex reasoning training: evidence from normal and clinical populations. Front Syst Neurosci.

[CR14] Chapman SB, Highley AP, Thompson JL (1998). Discourse in fluent aphasia and Alzheimer's disease: linguistic and pragmatic considerations. J Neurolinguistics.

[CR15] Chapman SB, Zientz J, Weiner M, Rosenberg R, Frawley W, Burns MH (2002). Discourse changes in early Alzheimer disease, mild cognitive impairment, and normal aging. Alzheimer Dis Assoc Disord.

[CR16] Chapman SB, Anand R, Sparks G, Cullum CM (2006). Gist distinctions in healthy cognitive aging versus mild Alzheimer's disease. Brain Impair.

[CR17] Chen P, Ratcliff G, Belle SH, Cauley JA, DeKosky ST, Ganguli M (2001). Patterns of cognitive decline in presymptomatic Alzheimer disease: a prospective community study. Arch Gen Psychiatry.

[CR18] Chenery HJ, Murdoch BE (1994). The production of narrative discourse in response to animations in persons with dementia of the Alzheimer's type: preliminary findings. Aphasiology.

[CR19] Chesneau S, Lepage É, Giroux F, Belleville S (2016). Trouble léger de la cognition: profils variés en compréhension de texte = Mild cognitive impairment: varied texts comprehension profiles. Revue canadienne d’orthophonie et d’audiologie = Can J Speech-Lang Pathol Audiol.

[CR20] Clarke N, Foltz P, Garrard P (2020). How to do things with (thousands of) words: computational approaches to discourse analysis in Alzheimer’s disease. Cortex.

[CR21] Cohen G (1979). Language comprehension in old age. Cogn Psychol.

[CR22] Creamer S, Schmitter-Edgecombe M (2010). Narrative comprehension in Alzheimer's disease: assessing inferences and memory operations with a think-aloud procedure. Neuropsychology.

[CR23] Cummings JL (2000). Cognitive and behavioral heterogeneity in Alzheimer’s disease: seeking the neurobiological basis. Neurobiol Aging.

[CR24] Cummings JL, Darkins A, Mendez M, Hill MA, Benson D (1988). Alzheimer's disease and Parkinson's disease: comparison of speech and language alterations. Neurology.

[CR25] Dalton SG, Hubbard HI, Richardson JD (2019) Moving toward non-transcription based discourse analysis in stable and progressive aphasia. In: Seminars in speech and language. Thieme Medical Publishers 10.1055/s-0039-3400990 PMC11363584 31869847

[CR26] Daneman M, Merikle PM (1996). Working memory and language comprehension: a meta-analysis. Psychon Bull Rev.

[CR27] Das N (2019). Cognitive training and transcranial direct current stimulation in mild cognitive impairment: a randomized pilot trial. Front Neurosci.

[CR28] DeKosky S (2003). Early intervention is key to successful management of Alzheimer disease. Alzheimer Dis Assoc Disord.

[CR29] DeTure MA, Dickson DW (2019). The neuropathological diagnosis of Alzheimer’s disease. Mol Neurodegener.

[CR30] Devanand DP (2008). Combining early markers strongly predicts conversion from mild cognitive impairment to Alzheimer's disease. Biol Psychiat.

[CR31] Drummond C (2019). Narrative impairment, white matter damage and CSF biomarkers in the Alzheimer's disease spectrum. Aging.

[CR32] Emery VOB (2000). Language impairment in dementia of the Alzheimer type: a hierarchical decline?. Int J Psychiatry Med.

[CR33] Fletcher CR, Chrysler ST (1990). Surface forms, textbases, and situation models: recognition memory for three types of textual information. Discourse Process.

[CR34] Folstein MF, Folstein SE, McHugh PR (1975). “Mini-mental state”: a practical method for grading the cognitive state of patients for the clinician. J Psychiatr Res.

[CR35] Fonseca R, Parente M, Côté H, Ska B, Joanette Y (2008). Introducing a communication assessment tool to Brazilian speech therapists: the MAC battery. Pro-fono.

[CR36] Galton CJ, Patterson K, Xuereb JH, Hodges JR (2000). Atypical and typical presentations of Alzheimer's disease: a clinical, neuropsychological, neuroimaging and pathological study of 13 cases. Brain.

[CR37] Gerwien J, von Stutterheim C (2018). Event segmentation: cross-linguistic differences in verbal and non-verbal tasks. Cognition.

[CR38] Graesser AC, Millis KK, Zwaan RA (1997). Discourse comprehension. Annu Rev Psychol.

[CR39] Graville DJ, Rau MT (1991). Reading comprehension of directly stated and inferred information in paragraph-length material by nondemented and demented elderly subjects clinical. Aphasiology.

[CR40] Haldenwanger A, Eling P, Kastrup A, Hildebrandt H (2010). Correlation between cognitive impairment and CSF biomarkers in amnesic MCI, non-amnesic MCI, and Alzheimer's disease. J Alzheimer's Dis.

[CR41] Henry JD, Crawford JR, Phillips LH (2004). Verbal fluency performance in dementia of the Alzheimer’s type: a meta-analysis. Neuropsychologia.

[CR42] Howieson DB, Dame A, Camicioli R, Sexton G, Payami H, Kaye JA (1997). Cognitive markers preceding Alzheimer's dementia in the healthy oldest old. J Am Geriatr Soc.

[CR43] Hughes CP, Berg L, Danziger W, Coben LA, Martin RL (1982). A new clinical scale for the staging of dementia. Br J Psychiatry.

[CR44] Huntley J, Howard R (2010). Working memory in early Alzheimer's disease: a neuropsychological review. Int J Geriatr Psychiatry.

[CR45] Imtiaz B, Tolppanen A-M, Kivipelto M, Soininen H (2014). Future directions in Alzheimer's disease from risk factors to prevention. Biochem Pharmacol.

[CR46] Jack CR (2018). NIA-AA research framework: toward a biological definition of Alzheimer's disease. Alzheimers Dement.

[CR47] Just MA, Carpenter PA (1992). A capacity theory of comprehension: individual differences in working memory. Psychol Rev.

[CR48] Kavé G, Levy Y (2003). Morphology in picture descriptions provided by persons with Alzheimer's disease. J Speech Lang Hear Res.

[CR49] Khoury R, Ghossoub E (2019). Diagnostic biomarkers of Alzheimer’s disease: a state-of-the-art review. Biomark Neuropsychiatry.

[CR50] Kintsch W (1988). The role of knowledge in discourse comprehension: a construction-integration model. Psychol Rev.

[CR51] Kintsch W, Rawson KA, Snowling MJ, Hulme C (2005). Comprehension. The science of reading: a handbook.

[CR52] Kintsch W, Van Dijk TA (1978). Toward a model of text comprehension and production. Psychol Rev.

[CR53] Kmet LM, Cook LS, Lee RC (2004) Standard quality assessment criteria for evaluating primary research papers from a variety of fields

[CR54] Laine M, Laakso M, Vuorinen E, Rinne J (1998). Coherence and informativeness of discourse in two dementia types. J Neurolinguistics.

[CR55] Lim YY (2020). Association of deficits in short-term learning and Aβ and hippoampal volume in cognitively normal adults. Neurology.

[CR56] Livingston G (2017). Dementia prevention, intervention, and care. Lancet.

[CR57] MacDonald MC, Almor A, Henderson VW, Kempler D, Andersen ES (2001). Assessing working memory and language comprehension in Alzheimer's disease. Brain Lang.

[CR58] Mazzon G (2019). Connected speech deficit as an early hallmark of CSF-defined Alzheimer's disease and correlation with cerebral hypoperfusion pattern. Curr Alzheimer Res.

[CR59] McKhann GM, Drachman D, Folstein M, Katzman R, Price D, Stadlan EM (1984). Clinical diagnosis of Alzheimer's disease: Report of the NINCDS-ADRDA Work Group* under the auspices of Department of Health and Human Services Task Force on Alzheimer's Disease. Neurology.

[CR60] McKhann GM (2011). The diagnosis of dementia due to Alzheimer's disease: recommendations from the National Institute on Aging-Alzheimer's Association workgroups on diagnostic guidelines for Alzheimer's disease. Alzheimer's Dement.

[CR61] Mudar RA, Nguyen LT, Eroh J, Chiang H-S, Rackley A, Chapman SB (2019). Event-related neural oscillation changes following reasoning training in individuals with mild cognitive impairment. Brain Res.

[CR62] Mueller KD, Hermann B, Mecollari J, Turkstra LS (2018). Connected speech and language in mild cognitive impairment and Alzheimer's disease: a review of picture description tasks. J Clin Exp Neuropsychol.

[CR63] Park DC, Reuter-Lorenz P (2009). The adaptive brain: aging and neurocognitive scaffolding. Annu Rev Psychol.

[CR64] Patterson C (2018). World Alzheimer report 2018: the state of the art of dementia research: new frontiers.

[CR65] Perneczky R, Wagenpfeil S, Komossa K, Grimmer T, Diehl J, Kurz A (2006). Mapping scores onto stages: mini-mental state examination and clinical dementia rating. Am J Geriatr Psychiatry.

[CR66] Petersen RC, Smith GE, Waring SC, Ivnik RJ, Tangalos EG, Kokmen E (1999). Mild cognitive impairment: clinical characterization and outcome. Arch Neurol.

[CR67] Petersen RC (2001). Current concepts in mild cognitive impairment. Arch Neurol.

[CR68] Pistono A, Jucla M, Bezy C, Lemesle B, Le Men J, Pariente J (2019). Discourse macrolinguistic impairment as a marker of linguistic and extralinguistic functions decline in early Alzheimer's disease. Int J Lang Commun Disord.

[CR69] Price BH, Gurvit H, Weintraub S, Geula C, Leimkuhler E, Mesulam M (1993). Neuropsychological patterns and language deficits in 20 consecutive cases of autopsy-confirmed Alzheimer's disease. Arch Neurol.

[CR70] Radvansky GA, Dijkstra K (2007). Aging and situation model processing. Psychon Bull Rev.

[CR71] Reilly J, Troche J, Grossman M, Budson AE, Kowall NW (2011). Language processing in dementia. The handbook of Alzheimer’s disease and other dementias.

[CR72] Rentz DM, Parra Rodriguez MA, Amariglio R, Stern Y, Sperling R, Ferris S (2013). Promising developments in neuropsychological approaches for the detection of preclinical Alzheimer’s disease: a selective review. Alzheimer's Res Ther.

[CR73] Rochon E, Waters GS, Caplan D (1994). Sentence comprehension in patients with Alzheimer′ s disease. Brain Lang.

[CR74] Sachdev PS, Blacker D, Blazer DG, Ganguli M, Jeste DV, Paulsen JS, Petersen RC (2014). Classifying neurocognitive disorders: the DSM-5 approach. Nat Rev Neurol.

[CR75] Schmidtke K, Hermeneit S (2008). High rate of conversion to Alzheimer's disease in a cohort of amnestic MCI patients. Int Psychogeriatr.

[CR76] Schmitter-Edgecombe M, Creamer S (2010). Assessment of strategic processing during narrative comprehension in individuals with mild cognitive impairment. J Int Neuropsychol Soc.

[CR77] Slegers A, Filiou R-P, Montembeault M, Brambati SM (2018). Connected speech features from picture description in Alzheimer’s disease: a systematic review. J Alzheimers Dis.

[CR78] Sparks JR, Seel NM (2012). Language/discourse comprehension and understanding. Encyclopedia of the sciences of learning.

[CR79] Stern Y (2012). Cognitive reserve in ageing and Alzheimer's disease. Lancet Neurol.

[CR80] Stern Y (2013). Cognitive reserve: implications for assessment and intervention. Folia Phoniatr Logop.

[CR81] Taler V, Phillips NA (2008). Language performance in Alzheimer's disease and mild cognitive impairment: a comparative review. J Clin Exp Neuropsychol.

[CR82] Thorndyke PW (1976). The role of inferences in discourse comprehension. J Verbal Learn Verbal Behav.

[CR83] Toledo CM, Aluísio SM, dos Santos LB, Brucki SMD, Trés ES, de Oliveira MO, Mansur LL (2018). Analysis of macrolinguistic aspects of narratives from individuals with Alzheimer's disease, mild cognitive impairment, and no cognitive impairment. Alzheimer's Dement.

[CR84] Tort-Merino A (2017). Early detection of learning difficulties when confronted with novel information in preclinical Alzheimer’s disease stage 1. J Alzheimer's Dis.

[CR85] Trabasso T, Magliano JP (1996). Conscious understanding during comprehension. Discourse Process.

[CR86] Ulatowska HK, Chapman SB, Highley AP, Prince J (1998). Discourse in healthy old-elderly adults: a longitudinal study. Aphasiology.

[CR87] Ulatowska HK, Chapman S, Johnson J, Branch C (1999). Macrostructure and inferential processing in discourse of aphasic patients. Psychol Lang.

[CR88] Van Dijk TA (2019). Macrostructures: an interdisciplinary study of global structures in discourse, interaction, and cognition.

[CR89] Vas AK, Spence J, Chapman SB (2015). Abstracting meaning from complex information (gist reasoning) in adult traumatic brain injury. J Clin Exp Neuropsychol.

[CR90] Vas AK, Spence JS, Eschler B, Chapman SB (2016). Sensitivity and specificity of abstraction using gist reasoning measure in adults with traumatic brain injury. J Appl Biobehav Res.

[CR91] Verma M, Howard RJ (2012). Semantic memory and language dysfunction in early Alzheimer's disease: a review. Int J Geriatr Psychiatry.

[CR92] Villemagne VL (2011). Longitudinal assessment of Aβ and cognition in aging and Alzheimer disease. Ann Neurol.

[CR93] Vuorinen E, Laine M, Rinne J (2000). Common pattern of language impairment in vascular dementia and in Alzheimer disease. Alzheimer Dis Assoc Disord.

[CR94] Ward A, Tardiff S, Dye C, Arrighi HM (2013). Rate of conversion from prodromal Alzheimer's disease to Alzheimer's dementia: a systematic review of the literature. Dement Geriatr Cogn Disord Extra.

[CR95] Welland RJ, Lubinski R, Higginbotham DJ (2002). Discourse comprehension test performance of elders with dementia of the Alzheimer type. J Speech Lang Hear Res.

[CR96] Winblad B (2004). Mild cognitive impairment—beyond controversies, towards a consensus: report of the International Working Group on Mild Cognitive Impairment. J Intern Med.

[CR97] Xu W (2015). Meta-analysis of modifiable risk factors for Alzheimer's disease. J Neurol Neurosurg Psychiatry.

