# Supplementary material for: Can discourse processing performance serve as an early marker of Alzheimer’s disease and mild cognitive impairment? A systematic review of text comprehension
Source: Eur J Ageing. 2021 Apr 20;19(1):3–18. doi: 10.1007/s10433-021-00619-5 (PMC8881530; doi:10.1007/s10433-021-00619-5)
Supplement: Supplementary file 2 — Supplementary file2 (DOCX 12 kb) [file 10433_2021_619_MOESM2_ESM.docx]

| **S2: Search strategy** | | | | |
| --- | --- | --- | --- | --- |
| **Database** | **Search string** | **Hits** | **Date** | **Years** |
| Web of Science | \|  \| (TS=("alzheimer's disease" OR "mild cognitive impairment") AND TS=(discourse) OR TS=("global coherence") OR TS=(macrolinguistic) OR TS=("connected language") OR TS=("connected speech") OR TS=("narrative comprehension") OR TS=("narrative speech")) *AND* **LANGUAGE:** (English) *AND* **DOCUMENT TYPES:** (Article)  Indexes=SCI-EXPANDED, SSCI Timespan=All years \| \| --- \| --- \| | 1597  253 | 08.03.2018  20.01.2020 | 1934-2020 |
| PubMed/ MedLine | (((((((("alzheimer's disease"[Title/Abstract] OR "mild cognitive impairment"[Title/Abstract]) AND discourse[Title/Abstract]) OR "global coherence"[Title/Abstract]) OR "narrative discourse"[Title/Abstract]) OR macrolinguistic[Title/Abstract]) OR "connected language"[Title/Abstract]) OR "narrative comprehension"[Title/Abstract]) OR "connected speech"[Title/Abstract]) OR "narrative speech"[Title/Abstract] | 955  180 | 08.03.2018  20.01.2020 | 1954-2020 |
| PsycINFO/ EBSCO | ("alzheimer's disease" OR "mild cognitive impairment") AND discourse OR "global coherence" OR "narrative discourse" OR macrolinguistic OR "connected language" OR "narrative comprehension" OR "connected speech" OR "narrative speech"  Language: English | 1587  144 | 08.03.2018  20.01.2020 | 1934-2020 |
